# A software-based tool for video motion tracking in the surgical skills assessment landscape

**DOI:** 10.1007/s00464-018-6023-5

**Published:** 2018-01-16

**Authors:** Sandeep Ganni, Sanne M. B. I. Botden, Magdalena Chmarra, Richard H. M. Goossens, Jack J. Jakimowicz

**Affiliations:** 1 Medisign, Industrial Design Engineering, Delft University of Technology, Delft, The Netherlands; 2 Department of Surgery, GSL Medical College, Rajahmundry, India; 3 Research and Education, Catharina Hospital, Michelangelolaan 2, 5653 EJ Eindhoven, The Netherlands; 4 Department of Pediatric Surgery, Radboudumc – Amalia Children’s Hospital, Nijmegen, The Netherlands

**Keywords:** Motion tracking, Objective assessment, Laparoscopic cholecystectomy, Laparoscopic skills, Training, Video-based assessment

## Abstract

**Background:**

The use of motion tracking has been proved to provide an objective assessment in surgical skills training. Current systems, however, require the use of additional equipment or specialised laparoscopic instruments and cameras to extract the data. The aim of this study was to determine the possibility of using a software-based solution to extract the data.

**Methods:**

6 expert and 23 novice participants performed a basic laparoscopic cholecystectomy procedure in the operating room. The recorded videos were analysed using Kinovea 0.8.15 and the following parameters calculated the path length, average instrument movement and number of sudden or extreme movements.

**Results:**

The analysed data showed that experts had significantly shorter path length (median 127 cm vs. 187 cm, *p* = 0.01), smaller average movements (median 0.40 cm vs. 0.32 cm, *p* = 0.002) and fewer sudden movements (median 14.00 vs. 21.61, *p* = 0.001) than their novice counterparts.

**Conclusion:**

The use of software-based video motion tracking of laparoscopic cholecystectomy is a simple and viable method enabling objective assessment of surgical performance. It provides clear discrimination between expert and novice performance.

Current training and evaluation in laparoscopic surgery require a combination of knowledge-based and technical skills assessment [1, 2]. Acquiring the necessary skills takes time, patience and technical aids, such as box trainers, virtual and augmented reality simulators [3–6]. One of the aims of these simulators is the attempt to reduce the reliance upon subjective expert observers when evaluating performance or assessing the acquisition of technical skills [7–10]. This is achieved by motion tracking of the instruments during the performance of, for example, laparoscopic cholecystectomy tasks and procedures in these simulated settings.

Motion tracking is a process where the location, movements, speed and/or acceleration of the instruments used by a surgeon are measured continuously whilst performing a procedure. Current tracking systems use different technologies (e.g. mechanical, optical, acoustic or electromagnetic) to collect the data about the instrument movements and forces applied. The instrument movements and applied forces are the parameters which are used to assess the performance by comparing them against a set of predetermined criteria [11].

The difficulty of extracting a set of criteria suitable for reliable, objective assessment of performance has been a significant challenge for these technical methods [12]. The specific difficulty is how to convert the measures recorded, including instrument position, path length, jerk index, speed, acceleration, etc., into a set of objective criteria which differentiates between competence and weaknesses. It has been shown that motion tracking can in fact be used to generate an objective set of criteria; this, however, necessitates a validation of expert performances to determine the benchmark for optimal performance. [13–16].

Current instrument tracking systems need additional equipment, or the use of special laparoscopic instruments, to facilitate the data acquisition and processing. Including additional recording equipment has not only the disadvantage of cost, but it can be very difficult to use in the clinical setting. Therefore, currently, assessments based on motion tracking are often done outside the clinical setting in a simulator (e.g. augmented or virtual reality simulator). Moreover, these methods only facilitate prospective analysis of surgical procedures and as such cannot be used for retrospective analysis. It would, however, be useful to have an automated tracking system to objectively assess the surgical skills in the clinical setting. Ideally, this tracking system would not require changing the currently used surgical instruments, analyse video recordings of procedures or selected component tasks without requiring pre-preparation, using little to none valuable space in the operating theatre and not hindering the ergonomics and safety of an already difficult form of surgery.

Thus, the aim of this study is to evaluate whether or not it is possible to extract a set of objective criteria from videos of conventional laparoscopic cholecystectomy, by means of motion tracking. For this, a dedicated software has been used to avoid the use of additional equipment in the operating room.

## Materials and methods

### Participants

All participants recruited for the study were either from Catharina Hospital, Eindhoven, The Netherlands (expert laparoscopic surgeons, who have performed more than 300 laparoscopic procedures) or the participants of the Laparoscopic Surgical Skills Curriculum Grade 1 Level 1 (surgical residents, who have performed fewer than ten laparoscopic procedures). There were 6 expert (over 300 procedures conducted) participants and 23 novice participants. All participants gave their consent for the video recording of them conducting the procedure to be used in this study and hospital ethics approval was obtained.

### Task description

The participants performed a basic laparoscopic cholecystectomy procedure in the operating room. For both expert and non-expert participants, all the patients operated on were uncomplicated cases without any contraindications. Non-expert participants who needed help from their instructor were excluded from the study. After the procedure, the videos were collected from the operating complex database.

### Data extraction

**Fig. 1:**
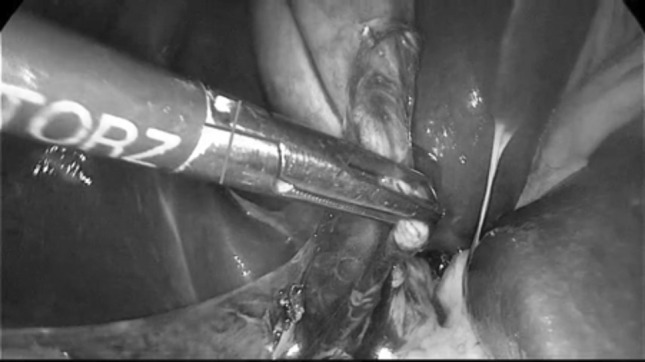
Image generated by Kinovea showing the placement of both the starting point (red cross) and the scale-lines (green and blue)

Raw video files of the clinical laparoscopic procedures were imported into Kinovea 0.8.15. Initial starting points were identified for three measurements: the first joint of the instrument, and two perpendicular lines, which were used for scale (see Fig. 1). Coordinate data (x,y) were extracted using the software in semi-automatic mode—that is, where a tracking point (or line) is placed and then the software attempts to automatically track where the same pixels are in the next frame. This placement was then manually checked and the locations adjusted in the cases where the software had not been able to correctly locate the same point(s) in consecutive frames.

### Camera distance

The videos were recorded with a laparoscopic camera during real laparoscopic procedures. During these procedures, an assistant operated the camera. Therefore, it was necessary to scale the (x,y) coordinate data using the (fixed, known) size of the surgical implement in the images. This was achieved by using the two perpendicular lines oriented along the second segment of the implement, from which, being of known length and angle within the image, an x- and y-scaling is calculated. This makes videos taken at different distances from the site of the operation easily comparable with each other. The coordinates were then adjusted using this scaling and converted to real-life distances based upon pixel width in the video ready for the calculation of statistics (Table 1). The limitation of this scaling is that the angle of the implement in the z-direction, may affect the result in a 2D video. This was overcome by using a pair of perpendicular lines for scaling.

**Table 1:** Criteria measured in the procedure and details their calculation

Description	Symbol	Formulae	Units
x-coordinate at frame n	${x_n}$		Pixels
y-coordinate at frame n	${y_n}$		Pixels
Number of frames	$N$		n/a
Number of frames per second	$f$		n/a
Scaled x-coordinate at frame n	$x_{n}^{'}$		cm
Scaled y-coordinate at frame n	$y_{n}^{'}$		cm
Distance moved between consecutive frames	${d_{n+1}}$	$\sqrt {{{\left( {x_{{n+1}}^{'} - x_{n}^{'}} \right)}^2}+{{\left( {y_{{n+1}}^{'} - y_{n}^{'}} \right)}^2}}$	cm
Total time taken	$T$	$\frac{N}{f}$	S
Total distance travelled	$D$	$\mathop \sum \limits_{{n=1}}^{{N - 1}} {d_{n+1}}$	cm
Average distance travelled per frame	$\overline {D}$	$\frac{D}{{N - 1}}$	cm
Average speed	$\overline {S}$	$\frac{D}{T}$	cm/s
Number of extreme movements	$M_{{n+1}}^{e}$	$$\left\{ {\begin{array}{*{20}{c}} 1&{{d_{n+1}} \geqslant {d_{th}}} \\ 0&{{d_{n+1}}<{d_{th}}} \end{array}} \right.$$	n/a
Number of extreme movements	$E$	$\mathop \sum \limits_{{n=1}}^{{N - 1}} M_{{n+1}}^{e}$	n/a

### Missing data points

Occasionally, the instrument had moved out of the field of view of the camera, or tissue was covering it. The software was able to automatically relocate the point in the majority of cases. In the former case, it was more difficult to relocate the same point for tracking as before (causing the wrong pixel to be tracked going forward). The results in this study were generated by making a decision on a case-by-case basis whether to approximate the location of the point (if enough of the instrument was visible to locate it manually) or to not track that section of the video. If the section was not counted, mean movement was considered to have happened between the last tracked point and the newly found point.

### Bias

Additionally, to assess the extent to which bias was a factor in this intervention, the statistics were calculated for a section of the videos of the surgical procedure where the instrument was always visible. Here, the algorithm was allowed to run completely automatically and the results compared with the semi-automatic procedure. These results were then compared with the overall results.

### Statistics

Three parameters were calculated: the path length, that is the total distance the tip of the instrument has travelled during the procedure; the average distance the instrument tip moved per time frame; and the number of extreme movements (defined as more than 1 cm movement per frame). Four other parameters were calculated from the extracted data using MATLAB (R16b), namely, (1) the Euclidian distance between each consecutive pair of points and (2) the average movement; the number of movements of a distance both (3) under, and (4) above a certain threshold. The statistics were presented using Graphpad Prism and, because the data were non-parametric, the Wilcoxon Signed-Rank test was used to calculate significant differences between the assessment scores. A *p* value of < 0.05 was considered statistically significant.

## Results

**Fig. 2:**
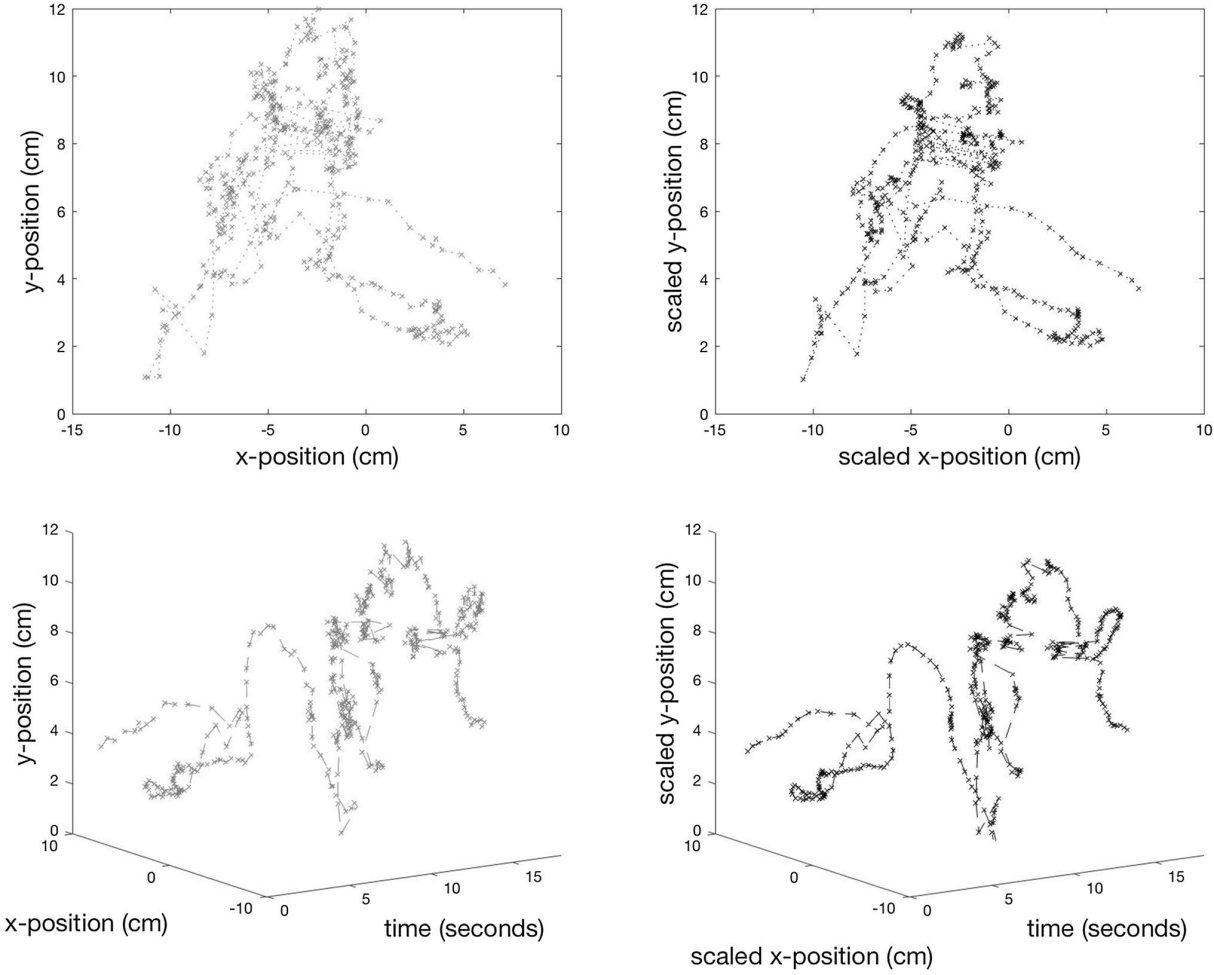
TOP ROW: A path, unscaled (x, y) for an 18-s period and BOTTOM ROW: the same path scaled for camera movement (x′ and y′). LEFT: 2-dimensional visualisation of the measurements; RIGHT: 3-dimensional visualisation (x(′), y(′) and time) of the measurements

In total, the data of 29 participants are included in this study, of which 6 were experts and 23 novices. An example of 18 s’ worth of output from the tracking for one instrument is shown in Fig. 2 with a 3D representation—x and y coordinates with time—to make the position of the implement clearer.

### Procedural results

**Fig. 3:**
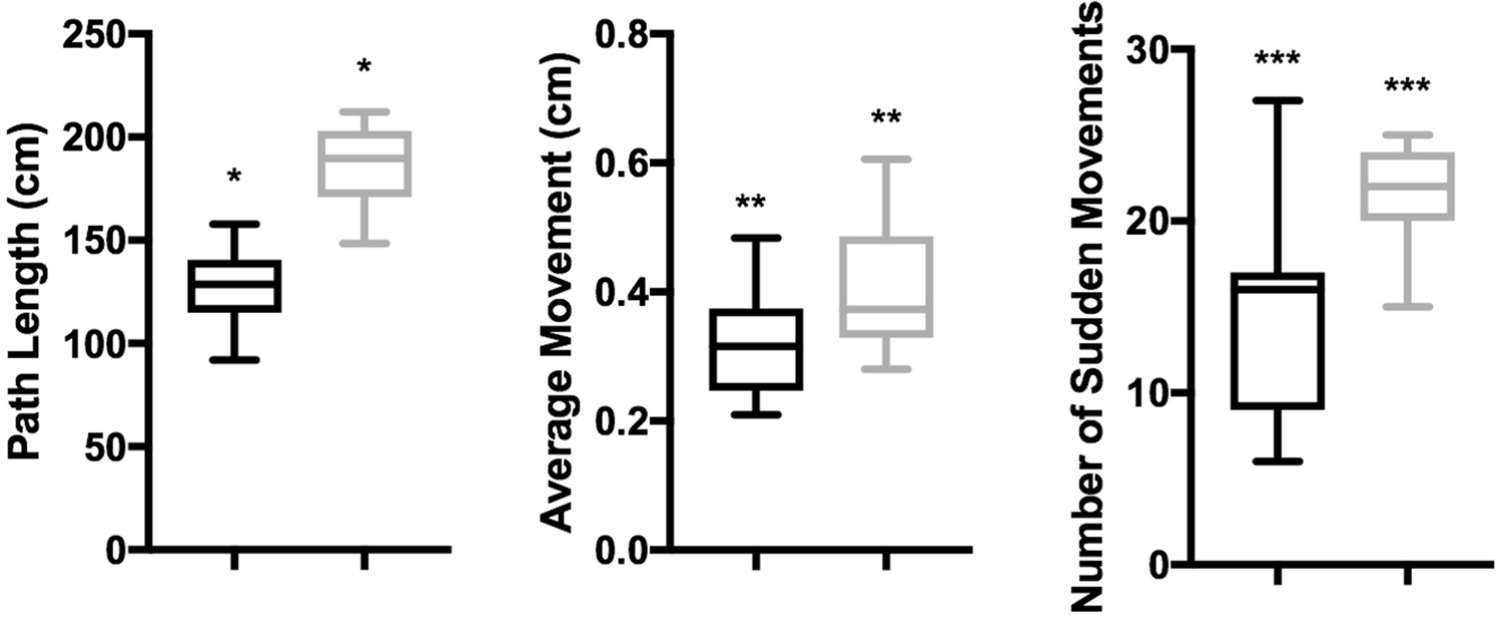
Summary data of the total path length, average movement and number of sudden movements (above 3.5 cm). Expert (black) and non-expert (grey) surgeons. *,** and *** indicate pairs of significant difference

The summary data for path length, average movement and the number of sudden movements are shown in the box-and-whisker plots in Fig. 3. Experts had significantly shorter path length (median 127 cm vs. 187 cm, *p* = 0.01), smaller average movements (median 0.40 cm vs. 0.32 cm, *p* = 0.002) and fewer sudden movements (median 14.00 vs. 21.61, *p* = 0.001) than their novice counterparts. No statistical difference was seen in path length per minute. (median 41.6 cm/min vs. 43.6 cm/min).

### Overcoming bias

**Fig. 4:**
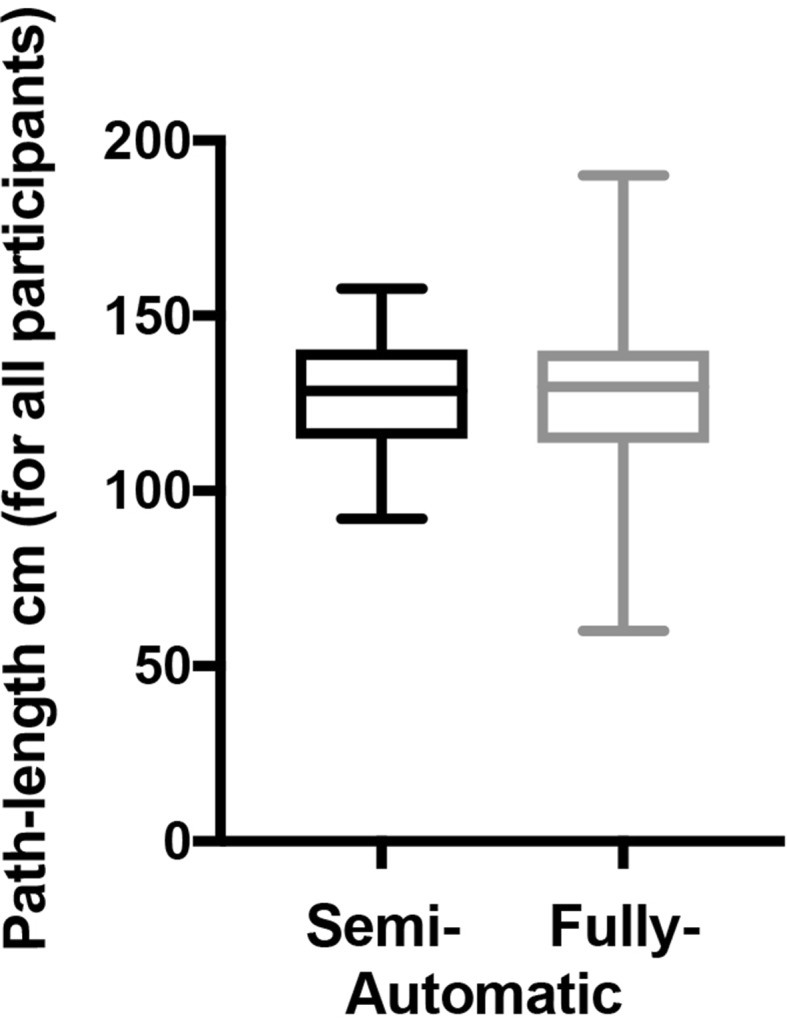
Comparison of spread of path length data using semi- (black) and fully automated (grey) procedure

As can be seen from Fig. 4, the median response was within 5% for the average distance travelled for each group but the spread of the data was increased by a couple of significant outliers. Manual analysis these outliers revealed that these were all caused by the wrong pixel being identified when the instrument re-entered the frame. It was therefore deemed necessary to manually identify the correct points in these cases.

## Discussion

The advantages of the ability to evaluate performance during laparoscopic procedures without the need for additional equipment are clear; because, this would allow for an objective clinical assessment. Furthermore, a software-based solution would allow for retrospective evaluation of surgical procedures.

This study aimed to see whether video-based motion tracking system is adequate in differentiating between expert and non-expert outcomes, when their clinical performances are evaluated on video, without any additional equipment installed. The use of this video motion tracking allows for a 2-D, x-y path projection of the 3-D location over time. From this, it is not only possible to extract the time of the procedure, but more importantly the specific movements of the instruments with average speed and number of ‘extreme’ movements that are made by a surgeon. Our results confirmed our hypothesis that in all three of these criteria, that experts took less time, had more efficient instrument motion and made fewer extreme movements than their non-expert counterparts. No difference was seen in the speed, however, which compliments the results shown in the study conducted by Kowalewski et al. [17]. Taken together, this suggests that it may be possible to discriminate between expert and non-expert participants using this method. It seems that this is the case and thus this system could be used as an alternative to clinically cumbersome and costly methods of motion tracking.

This type of retrospective analysis may provide a way for determining the level of performance in laparoscopic surgery in future. However, it is necessary to establish thresholds for safe performance in laparoscopic cholecystectomy. The next step in establishing this would be to determine a set of thresholds/criteria based on the data this study resulted in and validate these in a blinded fashion.

## Limitations

Difficulties and limitations of using video files were overcome in procuring the data that enable objective evaluation of performance. The process is currently only semi-automatic—both in terms of the tracking and, indeed, in deciding the ‘window of interest’ in terms of the relevant part of the surgical procedure. Furthermore, it was necessary to decide on a case-by-case basis what to do when either the camera’s view does not include the instrument’s tip (for instance, it is covered by tissue). In particular, it was necessary to consider the effect of the camera’s movement in relation to the instrument tip (in all three spatial dimensions) in calculating the average distance moved.

Whilst there is no perfect solution to this, our procedure was to use the known size of the instrument’s joints in the frame to calculate the relative size of each pixel and then scale by the average of this seems to be a fair compromise. If using this method prospectively, an expert camera driver could be used but, for retrospective use, ideally this process should be automated in the future. In spite of these difficulties, the results clearly discriminate between those procedures performed by expert and novice surgeons.

## Conclusion

This technical alternative to expert assessment in clinical practise could prove very valuable for the evaluation of surgical skills. Because no extra instruments or additives are needed, this motion tracking system is usable for all surgeons as an objective assessment of skills.

The use of video motion tracking of laparoscopic cholecystectomy is a simple and viable method enabling assessment of performance of the procedure. It provides clear discrimination between expert and novice performance.
